# Plastral Patterns Serve an Anti‐Predator Function in Freshwater Turtle Hatchlings

**DOI:** 10.1002/ece3.73913

**Published:** 2026-07-03

**Authors:** Morgan A. Clark, Claudia Crowther, Beth A. Reinke

**Affiliations:** ^1^ Kellogg Biological Station Michigan State University Hickory Corners Michigan USA; ^2^ Institute of Organismic and Molecular Evolution Johannes Gutenberg University Mainz Germany; ^3^ Department of Biology Northeastern Illinois University Chicago Illinois USA

**Keywords:** antipredator defense, coloration, painted turtle, patterning, signal

## Abstract

Color and pattern serve as a range of anti‐predator defense mechanisms across taxa, from crypsis to warning coloration, and these phenomena are well described across invertebrate and vertebrate systems. Coloration is often assumed to have a consistent anti‐predator function across life stages, but research conducted at a single life stage can yield valuable insight into the ecological and evolutionary significance of coloration across shifts in stage‐specific selection pressures. Painted turtles ( *Chrysemys picta* ) are so named for their striking coloration and patterning. In this study, we use simulated hatchling painted turtles to test the anti‐predator function of shell coloration on land. Using resin models, we demonstrate, for the first time, that plastral patterns have an anti‐predatory function in painted turtle hatchlings in conditions ecologically relevant to nest dispersal. Specifically, turtle models with simple patterns were predated significantly more than their complex‐patterned counterparts. This suggests that plastral pattern may serve as an anti‐predator strategy through background matching or disruptive coloration. These results provide evidence for the poorly understood functional importance of color and pattern variation in this species and demonstrate the need to test the roles of defensive coloration and patterning across life stages to better understand their ecological and evolutionary significance.

## Graphical abstract

Color and pattern serve as a range of anti‐predator defense mechanisms across taxa, from crypsis to warning coloration, and these phenomena are well described across invertebrate and vertebrate systems. Painted turtles ( *Chrysemys picta* ) are so named for their striking coloration and patterning. In this study, we use simulated hatchling painted turtles to test the anti‐predator function of shell coloration on land. Using resin models, we demonstrate for the first time that plastral patterns have an anti‐predatory function in painted turtle hatchlings in conditions ecologically relevant to nest dispersal.

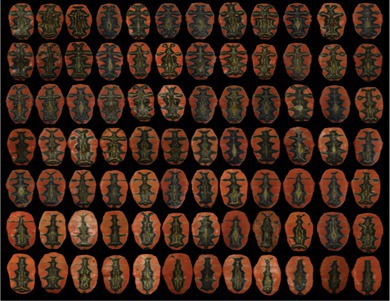

## 1 Introduction

Prey species across taxa exhibit a wide array of anti‐predator defense mechanisms, often producing striking behavioral, structural, and color variation (Arbuckle and Speed 2015; Kikuchi et al. 2023). In particular, the anti‐predator function of camouflaging or aposematic coloration and patterning has been well described (Stevens 2015). In many species, these anti‐predator defense mechanisms are known to shift ontogenetically within individuals as they experience changes in selective pressures across their lifetime (Barbosa et al. 2007; Despland 2020; Stückler et al. 2023; Wilson et al. 2007; Yuan et al. 2022). For organisms with complex and varied life history stages, the study of anti‐predator defense mechanisms at different ages can yield unique insight into the effect of defensive traits on lifetime fitness (Lindstedt et al. 2019). For example, assessing the covariation in snake color pattern and anti‐predator defense behaviors revealed that changes in behavior and color pattern across life stages work synergistically to increase fitness under different selection pressures (Creer 2005). Similarly, in alder moths, anti‐predator defense strategies switch from masquerade to conspicuous coloration across life stages, which promotes fitness as predation pressure changes across lifetime (Valkonen et al. 2014). Studying the ecological causes and consequences of anti‐predator coloration across life stages and taxa will allow us to better understand the general evolution of defensive traits and how these traits may influence broader species and population persistence under future change (Lindstedt et al. 2019).

The form and function of anti‐predator coloration and patterning are well described across invertebrate, fish, avian, and amphibian taxa (Götmark 1992; Hedley and Caro 2022; McLellan et al. 2023; Price et al. 2008; Santos et al. 2003; Sugiura 2020; Toledo and Haddad 2009) and in a limited number of reptilian groups, primarily snakes and lizards (Banci et al. 2020; Kikuchi et al. 2014; Miranda et al. 2023; Whiting et al. 2022). Often, assessments of anti‐predator coloration and pattern in these systems are assessed at the adult stage. The painted turtle ( *Chrysemys picta* ) is so named for its bright coloration and patterning, but the function of these traits is not entirely known (Reinke et al. 2017; Stasiek and Reinke 2025). Most work investigating the role of integumentary coloration in this species focuses on adults (Judson et al. 2026; Rowe et al. 2014; Stasiek and Reinke 2025; Steffen et al. 2019), yet painted turtle plastrons (ventral side of the shell) maintain variable red, orange, and black coloration and patterning from hatching. Variation in plastral coloration and pattern, and the ultimate ecological and evolutionary function of such variation, are underexplored, especially at the hatchling stage.

Painted turtles are a freshwater species widespread across North America and are a model system for understanding life‐history trade‐offs (Reinke et al. 2019; Valenzuela 2009), aging (Warner et al. 2016), behavior (Clark et al. 2025), and sex‐determining mechanisms (Bodensteiner et al. 2023; Refsnider and Janzen 2016). Painted turtles face unique predation pressures that vary by life stage: in the northern parts of their range, post‐hatching young overwinter in nest cavities, which are often depredated by foraging mesopredators and experience challenging winter conditions (Costanzo et al. 2004; Refsnider et al. 2015; Riley et al. 2014; Strickland and Janzen 2010). Post overwintering, young emerge the following spring and face high predation pressure from terrestrial and avian predators as they disperse toward water (Colbert et al. 2022). Once in water, many young are predated by fish, frogs, birds, mammals, and other turtles (Ernst and Lovich 2009). As adults, painted turtles are predated by raptors while basking, and adult females especially face the risk of direct predation by terrestrial mesopredators during the nesting season (Clark et al. 2025; Delaney et al. 2017, 2020). Despite the presence of unique bright plastral coloration and pattern present across such variable life stages, the role of these traits as anti‐predator defense mechanisms has received little empirical attention.

In adult painted turtles and related species, studies have investigated the role of coloration from a proximate perspective, describing drivers of integumentary pigment coloration of freshwater species with colorful body stripes or post‐orbital spots, like painted turtles and red‐eared sliders ( *Trachemys scripta* ) (Steffen et al. 2015, 2019), or the relationship between physiological health and coloration (Judson et al. 2026; Stasiek and Reinke 2025). In hatchling freshwater turtles, research on coloration has focused on the potential role of bright red and orange plastral colors as an anti‐predator aposematic strategy, yielding mixed results. One study found that bright plastral coloration in painted and red‐eared slider turtle hatchlings is correlated with defensive behaviors, reducing predation by largemouth bass ( *Micropterus salmoides* ) through associative learning (Britson and Gutzke 1993). In contrast, in a similar study, largemouth bass showed no color‐associated learning in a painted turtle predation experiment (Britson 1998). Further, Reinke et al. (2017) tested the terrestrial predator perception ability of plastral colors and predation of clay hatchling models with different coloration and pattern treatments. Predators in this study could distinguish bright plastral coloration from the background, yet neither coloration nor pattern presence served an anti‐predator function (Reinke et al. 2017).

**FIGURE 1:**
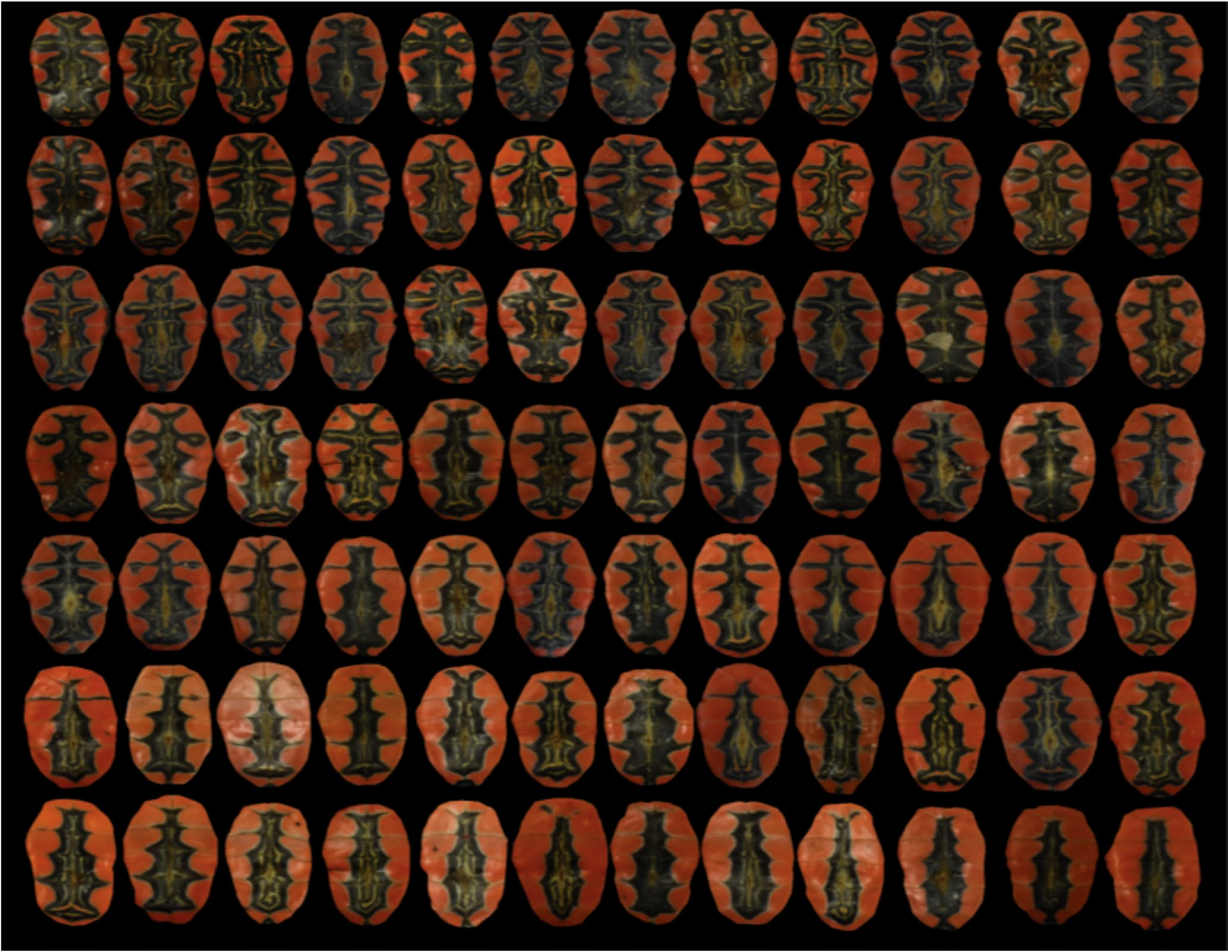
Plastral pattern variation in hatchling painted turtles collected from Thomson Causeway Recreation Area, adjacent habitat to where the model hatchlings were deployed.

While the role of plastron color as an aposematic strategy has been tested, the function of the black plastral patterning, which is highly variable in complexity across individuals (Figure 1), has yet to be assessed. Reinke et al. (2017) found that the presence of a simple black plastral pattern did not significantly influence predation on models with and without bright coloration, yet models with black patterns trended toward being predated less frequently than those with patterns. This black plastral pattern is common and conserved across life stages (Cooley et al. 2013). Weighing on average less than 7 g each, hatchling turtles dispersing overland from the nest encounter frequent obstacles that lead them to flip ventral side up (all authors, personal observation; Steyermark and Spotila 2001). At this field site, the average painted turtle nest distance from water is 30 m, providing ample opportunity for tiny hatchlings to get snagged on rocks, dirt, and vegetation and become ventrally exposed (Delaney et al. 2020). Given that the orange plastral background coloration highly contrasts with natural nesting backgrounds as viewed by common predators (Reinke et al. 2017), and that the orange coloration also highly contrasts with the plastral pattern, the pattern may instead serve to disrupt the coloration and allow for better crypsis when a hatchling turtle is flipped on its carapace during dispersal from the nest. Disruptive coloration is defined by Stevens and Merilaita (2009) as markings that create the appearance of false edges and hinder the detection of an organism's true shape. These markings may be highly contrasting with other colors on the organism and may or may not touch the margins of the organism (Stevens and Merilaita 2009). It is unknown whether or how much the shape or size of the highly contrasting marks matters, and this may vary across species (Stevens and Merilaita 2009).

In this study, we used realistic model replicas of hatchling painted turtles, deployed in natural painted turtle nesting habitat, to test the anti‐predatory signaling function of black plastral patterns. Using these models, created with realistic plastral reflectance properties, we assessed hatchling model disturbance by predators using models with simple or complex plastral patterns, further elucidating the ecological and evolutionary drivers of color and pattern variation in this system. We predict that simple plastral patterns are less functional as disruptive coloration because they don't disrupt the overall shape of the turtle, and so we should see more predation attempts on turtle models with simple plastral patterns than on those with complex plastral patterns.

## 2 Methods

### 2.1 Model Making

**FIGURE 2:**
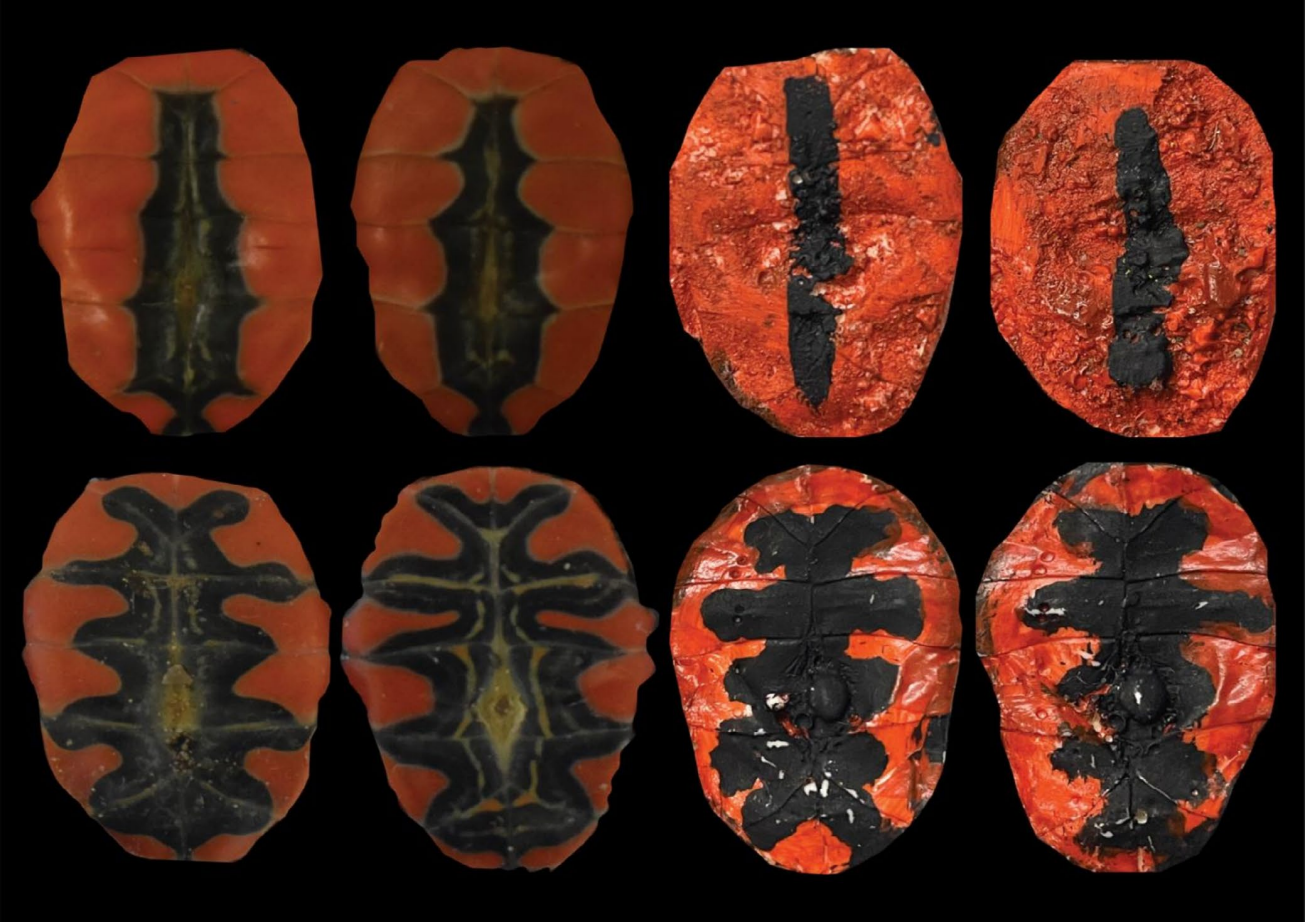
Simple (top) and complex (bottom) plastral patterns of real hatchlings (left) and model hatchlings (right).

We used a deceased *C. picta* hatchling preserved in ethanol to create molds using Silicone (BBDINO Super Elastic Platinum Silicone Rubber, Temple City, CA, USA). We then used the molds to cast 300 model turtles using polyurethane casting resin (Specialty Resin and Chemical Model‐Pro, Kalamazoo, MI, USA) in a vacuum chamber. We painted the replicas with paint colors with spectra matched to the colors of hatchling painted turtles (see reflectance spectra and paint matching below). Half of the models were painted with a simple line pattern, and half with a more complex pattern, both modeled after a real hatchling plastron (see Figure 2), hereafter referred to as the simple and complex models, respectively. We determined that the paint could not be removed by moderate friction but would chip under similar pressure when tapped by a fingernail (simulating an avian beak or claw). We also tested the durability of the paint under running tap water to assess if rainfall could alter the paint on models. After subjecting test models to low, medium, and high‐water flow from the faucet, we determined that water did not alter the paint, even under high pressure. We labeled the models with unique ID numbers on their carapaces so they could be identified in the array.

### 2.2 Reflectance Spectra and Paint Matching

To ensure the turtle models appeared as realistic as possible to all potential predator visual systems, we collected reflectance spectra from three live *C. picta* hatchlings from the field site. We measured the reflectance of the orange “background” color on the plastron, the black on the plastral shape, and the olive color on the carapace. Spectra were collected using an Ocean Optics Flame Spectrometer (Orlando, FL, USA) with a full‐spectrum light source and a bifurcated fiber optic probe. Reflectance was set relative to a 99% white reflectance standard. Negative values were zeroed, and spectra were smoothed using the *pavo* package (Maia et al. 2013) in R version 4.5.2 (2025‐10‐31). We then tested various acrylic paints and paint mixes on resin until we were able to match spectra of the dried paint samples to within the maximum standard deviation at any given wavelength that was observed across the three hatchlings for a given color patch (Figure S1). All live animal handling was approved by the Institutional Animal Care and Use Committee (IACUC) at Michigan State University (protocol number PROTO202300095).

### 2.3 Array Location and Design

Our study was conducted in the Thomson Sand Prairie, Thomson, Illinois, which is a known nesting ground for painted turtles (Mitchell et al. 2013; Shortridge et al. 2025; personal observation, all authors). Known turtle avian and terrestrial predators move through and forage at this site. In May 2024, we established 5, 25 m transects, spaced evenly 3 m apart, situated parallel to the Mississippi River (Figure S4). We deployed a motion‐activated camera trap at both ends of each transect, ~30 cm above the ground mounted to stakes pointing inward along the transect (*n* = 10, Blaze Video 64 MP Game & Trail Cameras). Cameras were equipped with night vision and were kept on continuously throughout the study, with photos offloaded daily to ensure memory space. Twenty hatchling models (10 complex, 10 simple) were arranged along each transect at 1 m intervals, placed plastral side up, and ordered randomly with respect to treatment group, for a total of 100 models. We inspected the array daily for 5 days at approximately midday, when predator activity was likely to be low. We scored the models for disturbance using the following rating system: 0 = no disturbance, 1 = movement but no damage, 2 = damage but no movement, 3 = movement and damage, 4 = flipped, 5 = destroyed or missing. We qualified damage as paint chipped or missing from the model, and destroyed if the model resin was broken or crushed in any way. Damaged models were replaced with a new model of the same pattern type.

#### 2.3.1 Camera Trap Analysis

Camera traps were used in this study to make a qualitative assessment of species that may prey on *C. picta* hatchlings, rather than a quantitative assessment of predation pressure. We manually reviewed all images captured by our camera traps.

### 2.4 Statistical Analysis

We modeled the effect of plastral pattern on replica hatchling disturbance using a generalized linear mixed model with a negative binomial probability distribution and log link. Our response variable was predation score (ranging from 0 to 5, see above) and our predictor was pattern complexity (categorical simple or complex). Precipitation was variable in the array during the course of the study, so we included binary presence of rainfall as a fixed effect in our model to ensure that it did not contribute to the difference in predation between model patterns. We also included the transect and position within the transect as random effects in the model. We used bootstrapped *p*‐values (boot = 1000) to assess the significance of fixed and random effects. We used the R package “DHARMa” to simulate residuals to test model fit, homogeneity of residuals, and dispersion (Hartig and Hartig 2017). All analyses and data visualization were conducted in R (R Core Team 2025). Figures were created with R package ggplot2 (Wickham 2016), grid, and gridExtra (Auguie 2017).

## 3 Results

### 3.1 Predation Pressure

**FIGURE 3:**
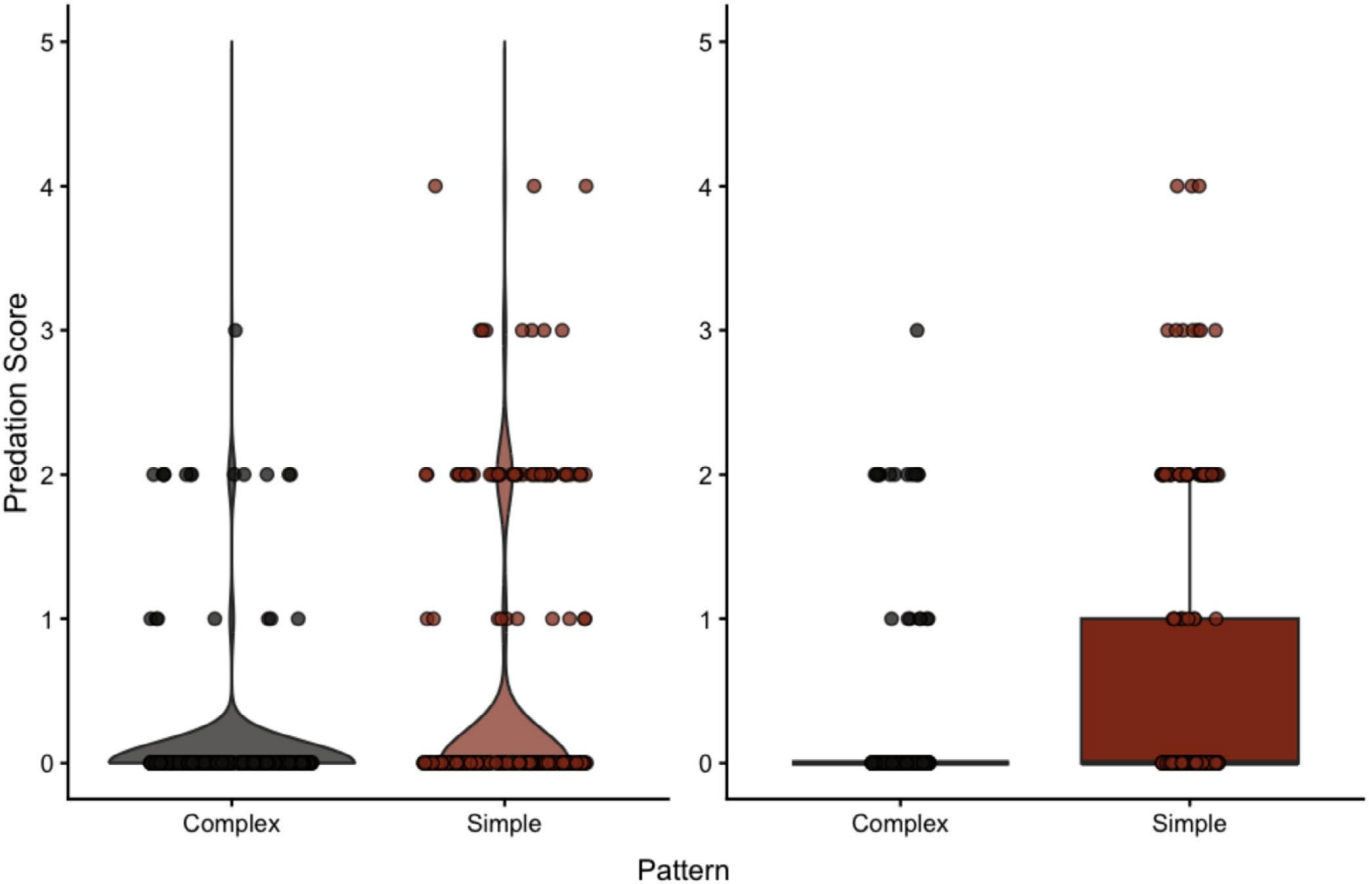
Turtle models with simple plastral patterns suffered significantly greater predation than their complex counterparts.

Increased plastral pattern complexity was associated with reduced risk of predation (*p* = 0.001, Figure 3, Table 1, Figure S2). Models with simple plastral patterns were over four times more likely to be damaged than models with complex patterns (10.8% of simple models vs. 2.6% of complex models). Simple and complex models scored in the movement only category at similar rates (2% of simple models vs. 1.4% of complex models).

**TABLE 1:** Results of analyses on predation by pattern, with an effect of rainfall.

Fixed effects	Coefficient estimate	Standard error	95% CI	*z* value	*p*
Intercept	−2.50	0.33	−3.15, −1.86	−7.587	
Pattern	1.34	0.27	0.82, 1.87	5.015	0.001
Rainfall	0.55	0.23	0.09, 1.00	2.345	0.010

Random effects	Standard deviation of random intercepts	*p*
Transect	0.16	0.159
Position	0.52	0.002

Models were more likely to be moved or damaged on days when rainfall occurred (12% without rainfall vs. 20% with rainfall, *p* = 0.010, Table 1), but rainfall had no significant interaction with predation score by pattern (*p* = 0.34, Table S1), i.e., the difference in predation risk between the patterns was not associated with rainfall (Table S3).

The random intercepts for the five transects were not significantly different (*p* = 0.159). However, position within the transect introduced ±0.52 SD to the log odds of predation score (*p* = 0.002, Table 1).

### 3.2 Observations of Potential Predators

**FIGURE 4:**
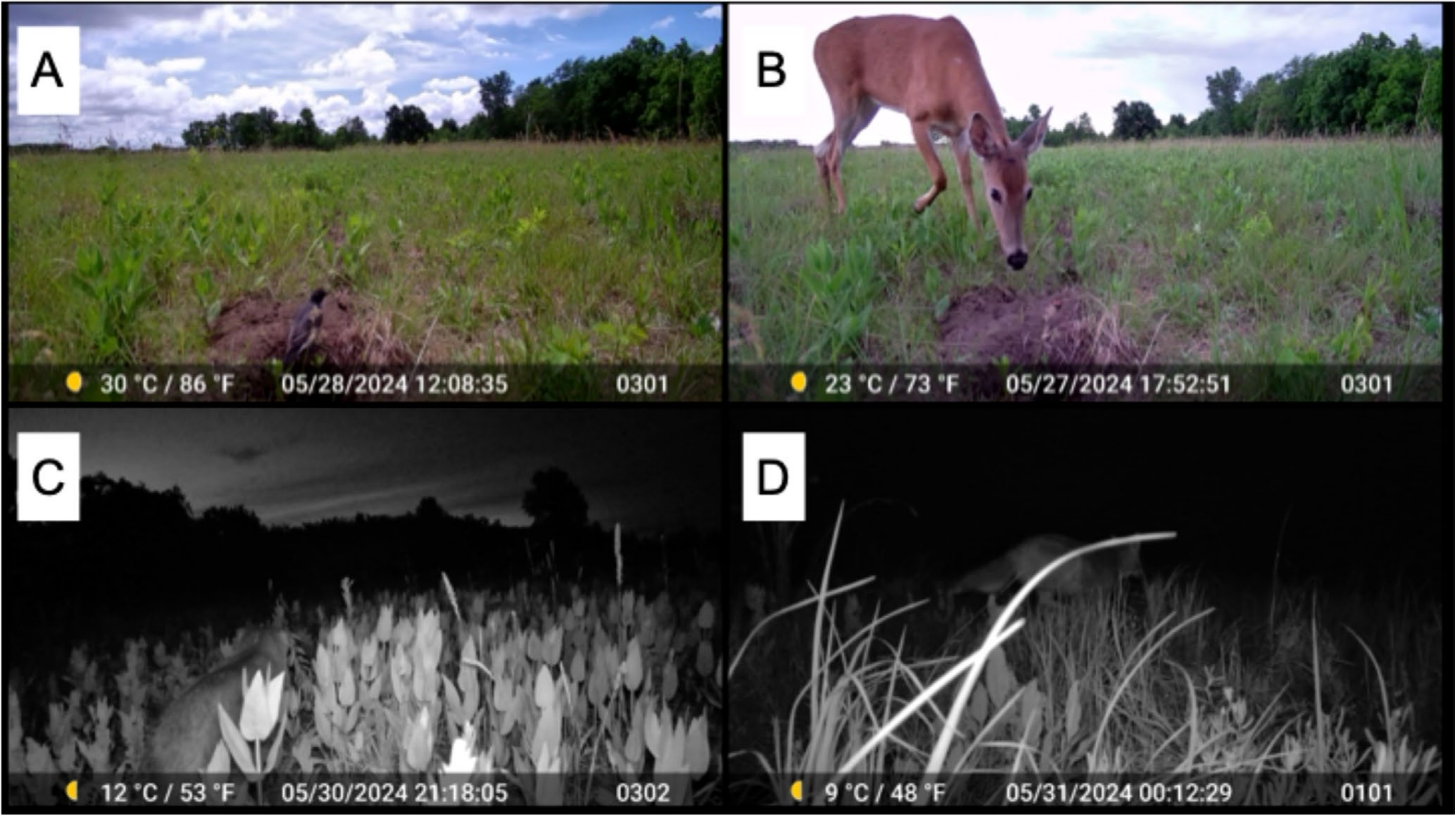
Potential predators interacting with model hatchling turtles. (A) Eastern Kingbird ( *Tyrannus tyrannus* ). (B) White‐tailed deer ( *Odocoileus virginianus* ), (C) Unknown mammal, (D) Fox (most likely *Vulpes vulpes* ).

Through our camera traps, we observed Eastern Kingbirds ( *Tyrannus tyrannus* ), white‐tailed deer ( *Odocoileus virginianus* ), a fox (most likely *Vulpes vulpes* ), and an unknown mammal (Figure 4) interacting with our models. Many mammals, including foxes, are known predators of hatchling *C. picta* (Ernst and Lovich 2009). Mammalian predators that are known to be in the area include raccoons, skunks, opossums, muskrats, minks, and otters. While white‐tailed deer have not been documented as predators of hatchling turtles, they have been known to eat eggs, fish, birds, snakes, and small mammals; so we cannot rule them out as a potential predator (Furness 1988; Pietz and Granfors 2000; Vazquez et al. 2023). Similarly, Eastern Kingbirds primarily feed on insects (including cicadas which are approximately the same size as hatchling turtles), but their sister species, Western Kingbirds ( *Tyrannus verticalis* ), have recently been documented to eat frogs as well (Titus 2024). Though we cannot determine for sure if the camera traps captured any true predation attempts, they still provide valuable qualitative data. The camera traps were only able to capture movement around models on the end of transects and so do not represent the only animals interacting with them.

## 4 Discussion

In this study, we used a painted turtle system to test the antipredator function of plastral shell coloration and pattern in an understudied life stage. By simulating hatchling painted turtles using resin models painted with simple and complex black plastral pattern types, we demonstrate for the first time in this system that plastral pattern complexity affects predation in conditions ecologically relevant to dispersal from the nest. Turtle models with simple plastral patterns suffered significantly greater predation than their complex counterparts (Figure 3). Until now, the adaptive significance of the black plastral pattern overlaying bright plastral coloration was unknown.

Previous work on painted turtle coloration tested the aposematic function of bright plastral color, concluding that this characteristic likely does not function as an anti‐predator signal (Britson 1998; Britson and Gutzke 1993; Reinke et al. 2017). Instead, bright plastral coloration may be a product of excess carotenoid deposition, where pro‐oxidants can be stored safely in shell tissue without causing physiological damage (Reinke et al. 2017). This may in part explain why some subspecies of painted turtles maintain bright coloration across life stages while others don't, if stored carotenoids aid in physiological freeze and anoxia tolerance (Reinke et al. 2017). Black plastral markings could create the appearance of false boundaries and edges of the overturned hatchling, ultimately decreasing predation by hindering predator recognition of prey (sensu Stevens and Merilaita 2009). For example, increased size of black spots on clay poison frog models decreased predation, despite their bright background colors (Preißler and Pröhl 2017). In a test with human subjects, simulated moths with a high number of disruptive edge patches were more difficult to detect and had higher survival, despite a lack of background matching (Webster et al. 2013). The complex plastral patterns simulated in the model painted turtle hatchlings used in this study could have created a disruptive visual effect, making the general outline of the hatchling more difficult to recognize as a prey item, thereby reducing depredation.

Additionally, if hatchling painted turtles have bright orange coloration that serves a physiological function, a more complex black pattern overlaying the plastron would reduce the area of bright coloration that is visually perceptible and may increase the degree of background matching with the substrate. Given that the bright plastral coloration in this species doesn't serve an anti‐predator aposematic function, it is possible that black pigments deposited over bright colors serve both to obscure the physiologically necessary bright plastral color and to disrupt the outline of the shell. Models with simple black patterns had more bright plastral color exposed, making them easier for predators to differentiate and locate against their background, while those with complex black patterns had less bright coloration visible and obscured edges. We didn't quantify the degree to which black plastral color matches with the substrate; future work in this system could test the degree of background matching supplied by black patterning using different predator visual modeling to directly test this hypothesis.

Likely the role of color and pattern in freshwater turtle species varies with changes in selective pressures across a lifetime. For example, plastral brightness in Diamondback terrapins ( *Malaclemys terrapin* ) varies by turtle sex and age; female terrapins lose plastral pigmentation as they increase in size over time and no longer experience predation risk from aquatic gape‐limited predators, forgoing the need for color‐based camouflage (Reinke et al. 2018). Similarly, some populations of green sea turtle ( *Chelonia mydas* ) hatchlings begin life with bright white plastral coloration, which serves as countershading in a pelagic environment, but these plastrons fade to gray or black during growth and as the turtles are released from predation pressure by small predators (Balazs 1986). Further, many hatchlings of Emydid turtles, including *Trachemys scripta* , *Clemmys guttata* , *Actinemys marmorata* , *Emydoidea blandingii* , and *Graptemys ouatchitensis*, have plastrons that contain bright colors with overlayed highly contrasting patterns. Whether the black plastral pattern of these other species has a similar antipredator function as we have found in the painted turtle remains to be seen. However, these colors and patterns frequently change or diminish with age; for example, the spotted turtle ( *Clemmys guttata* ) has a central dark pattern similar to *C. picta* in the hatchling stage, but this pattern grows with age until the plastron is almost entirely covered (Gray 2008). Similarly, *Trachemys scripta* has widely documented ontogenetic melanism of not only the plastron, but also the head and carapace, with a function and/or physiological basis that remains poorly understood (Lovich et al. 1990). This ontogenetic change in plastron pattern could represent a release from predation pressure.

In this study, the hatchling resin models were deployed ventral side up, to simulate an overturned hatchling during dispersal from the nest. While the plastral color and pattern of models in this study provide an ecologically relevant cue, they were exposed ventrally for a much longer period of time than real hatchlings would be in the wild. If live hatchlings were continuously exposed to the differential predation by plastral pattern that we document here, we would expect to see a population‐level decrease in the number of individuals with simple plastral patterns and a decrease in plastral pattern variation over time. However, this is not the case, as clutches from numerous mothers collected from this study area in recent years display variation in plastral pattern complexity (Figure 1). Furthermore, black plastral patterning and complexity vary geographically across painted‐turtle subspecies (all authors, personal observation). Future studies should assess the link between hatchling dispersal survivorship and plastral pattern across geographic regions and under varying selective pressures to validate the results we document here and provide insight into the ontogenetic adaptive significance of this trait in the wild.

Lastly, there was considerable variability in predation score between model positions within transects. It is possible that predators repeatedly use the same paths to cross prairie grassland where models were deployed to reach prey‐rich ecological edges, such as the riverbank, and, as a result, encounter models in the same array position each night. This is consistent with literature describing the foraging path patterns of potential terrestrial predators, such as raccoons (Newbury and Nelson 2007). However, this would be less ecologically relevant for avian predators. Models found to be damaged during a survey transect were replaced with a new model of the same pattern, and overall, the distribution of simple and complex models along the transect was randomly selected. The most common types of model disturbance observed were model displacement from its deployed location along the transect and small chips to the model paint, consistent with what would be produced by avian predation. Future studies using the same design may want to include a direct assessment of predator foraging paths to account for the potential effect of repeated foraging patterns across space and time.

The role of anti‐predator coloration and pattern is well documented across many invertebrate and vertebrate taxa, and assessing the role of coloration across different life stages can yield better insight into their ecological and evolutionary importance (Caro and Koneru 2021; Lindstedt et al. 2019; Postema et al. 2023). Using resin models, our study provides evidence for an anti‐predator function of the plastral pattern in hatchling painted turtles, an understudied life stage in turtle coloration research. This work is the first to demonstrate that variation in black plastral pattern may have fitness consequences during overland dispersal in this system. This work contributes to our growing understanding of the striking color and pattern polymorphisms present in a model taxon with high selection pressure during dispersal. As the understanding of protective coloration expands, future studies should test hypotheses across life stages to investigate the ecological and evolutionary drivers of defensive trait variation and their significance across diverse contexts.

## Author Contributions

**Morgan A. Clark:** conceptualization (equal), data curation (equal), investigation (equal), methodology (equal), project administration (equal), resources (equal), validation (equal), writing – original draft (lead), writing – review and editing (lead). **Claudia Crowther:** conceptualization (equal), data curation (equal), formal analysis (lead), funding acquisition (equal), investigation (equal), methodology (equal), project administration (equal), resources (equal), supervision (equal), validation (equal), visualization (lead), writing – review and editing (equal). **Beth A. Reinke:** conceptualization (equal), data curation (equal), funding acquisition (equal), investigation (equal), methodology (equal), project administration (equal), resources (equal), supervision (equal), validation (equal), writing – review and editing (equal).

## Conflicts of Interest

The authors declare no conflicts of interest.

## Supporting information


**Figure S1:** The reflectance spectra of turtle models (solid lines) were within a maximum standard deviation of the spectra of turtle hatchlings (dotted lines) for the orange “background” color, the black plastral pattern, and the olive‐colored carapace.
**Figure S2:** Turtle models with simple plastral patterns were predated more intensely than those with complex patterns.
**Figure S3:** Hatchling models were more likely to be predated during rainfall events, but the presence of rainfall did not drive a difference in predation between pattern types.
**Figure S4:** Google Earth image of the Thomson Sand Prairie, Thomson, Illinois. (A) Wide view of the sand prairie pictured right, and back‐channel slough of the Mississippi River pictured left. Black star indicates known nesting area for painted turtles. (B) Orientation of five, 25‐m‐long transects parallel to the water's edge. (C) Picture of the array. Each transect end was marked with a stake with an attached camera trap.
**Table S1:** The effect of pattern on turtle model predation with and without the effects of rainfall.

## Data Availability

The data that support the findings of this study are available in a Dryad Repository at the following link: https://doi.org/10.5061/dryad.qbzkh18z3.
