# Supplementary material for: Plastral Patterns Serve an Anti‐Predator Function in Freshwater Turtle Hatchlings
Source: Ecol Evol. 2026 Jul 3;16(7):e73913. doi: 10.1002/ece3.73913 (PMC13331868; doi:10.1002/ece3.73913)
Supplement: Supplementary file 1 — Figure S1: The reflectance spectra of turtle models (solid lines) were within a maximum standard deviation of the spectra of turtle hatchlings (dotted lines) for the orange “background” color, the black plastral pattern, and the olive‐colored carapace. Figure S2: Turtle models with simple plastral patterns were predated more intensely than those with complex patterns. Figure S3: Hatchling models were more likely to be predated during rainfall events, but the presence of rainfall did not drive a difference in predation between pattern types. Figure S4: Google Earth image of the Thomson Sand Prairie, Thomson, Illinois. (A) Wide view of the sand prairie pictured right, and back‐channel slough of the Mississippi River pictured left. Black star indicates known nesting area for painted turtles. (B) Orientation of five, 25‐m‐long transects parallel to the water's edge. (C) Picture of the array. Each transect end was marked with a stake with an attached camera trap. Table S1: The effect of pattern on turtle model predation with and without the effects of rainfall. [file ECE3-16-e73913-s001.docx]

SUPPLEMENTARY MATERIALS


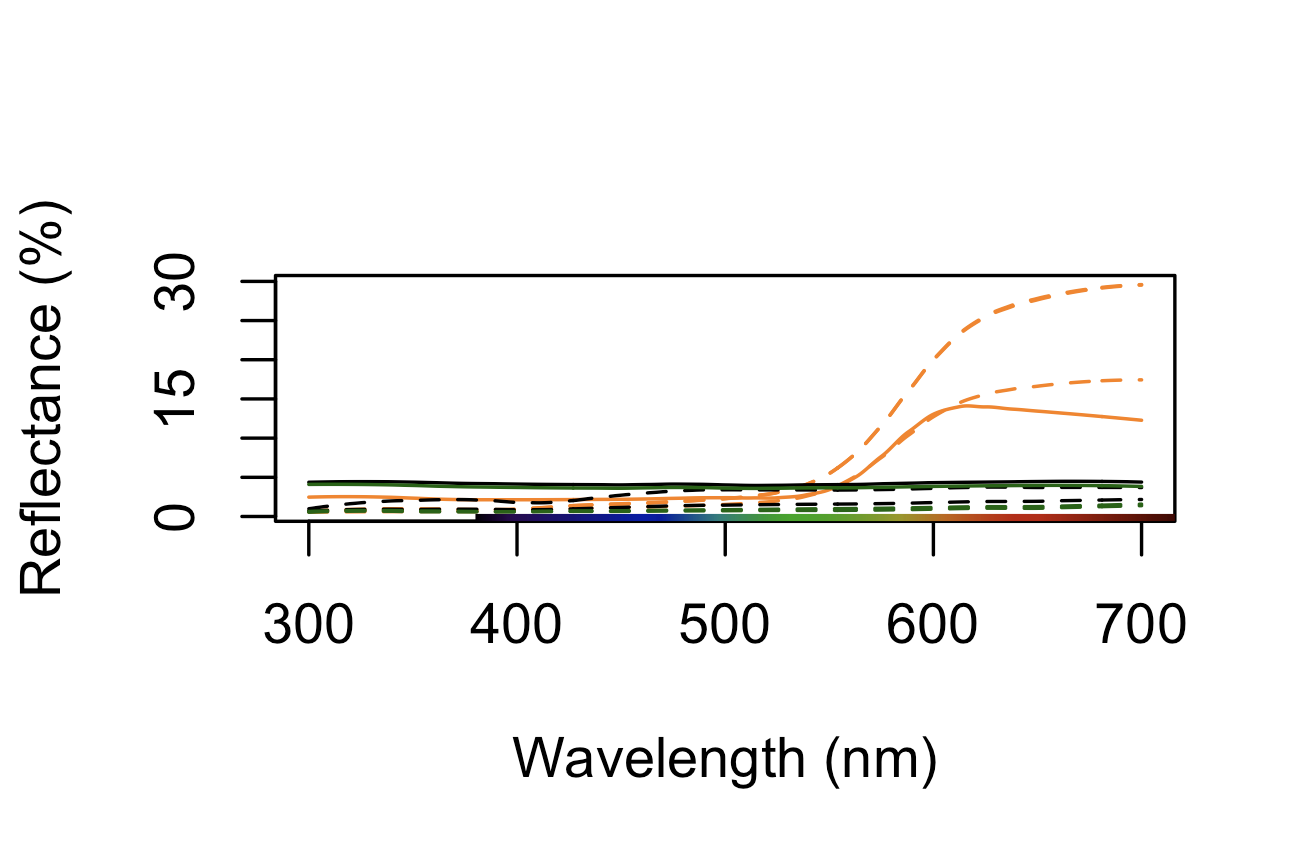


Supplementary Figure 1: The reflectance spectra of turtle models (solid lines) were within a maximum standard deviation of the spectra of turtle hatchlings (dotted lines) for the orange “background” color, the black plastral pattern, and the olive-colored carapace

Supplementary Figure 2: Turtle models with simple plastral patterns were predated more intensely than those with complex patterns

Supplementary Figure 3: Hatchling models were more likely to be predated during rainfall events, but the presence of rainfall did not drive a difference in predation between pattern types.

Supplementary Figure 4: Google Earth image of the Thomson Sand Prairie, Thomson, Illinois. Panel A: Wide view of the sand prairie pictured right, and back-channel slough of the Mississippi River pictured left. Black star indicates known nesting area for painted turtles. Panel B: Orientation of five, 25-meter-long transects parallel to the water’s edge. Panel C: Picture of the array. Each transect end was marked with a stake with an attached camera trap.

Supplementary Table 1: The effect of pattern on turtle model predation with and without the effects of rainfall

| **Model 1** | **Fixed Effects** | **Coefficient Estimate** | | | **Standard Error** | **95% CI** | **Z - value** | **P - value** | **df** | **AIC Value** |
| --- | --- | --- | --- | --- | --- | --- | --- | --- | --- | --- |
|  | Intercept | -2.1356 | | | 0.2850 |  | -7.494 |  | 495 | 667.9643 |
|  | Pattern | 1.3388 | | | 0.2681 |  | 4.994 | 5.91e-07 |  |  |
|  | **Random Effects** | **Standard deviation of random intercepts** | | | |  |  |  |  |  |
|  | Transect | 0.1707 | | | |  |  |  |  |  |
|  | Position | 0.5140 | | | |  |  |  |  |  |
| **Model 2** | **Fixed Effects** | **Coefficient Estimate** | | **Standard Error** | | **95% CI** | **Z - value** | **P - value** | **df** | **AIC Value** |
|  | Intercept | -2.8532 | | 0.5296 | |  | -5.387 |  | 493 | 665.0957 |
|  | Pattern | 1.7755 | | 0.5501 | |  | 3.228 | 0.00125 |  |  |
|  | Rainfall | 1.0080 | | 0.5573 | |  | 1.809 | 0.07049 |  |  |
|  | Rainfall*Pattern | -0.5765 | | 0.6139 | |  | -0.939 | 0.34771 |  |  |
|  | **Random Effects** | **Standard deviation of random intercepts** | | | |  |  |  |  |  |
|  | Transect | 0.1615 | | | |  |  |  |  |  |
|  | Position | 0.5196 | | | |  |  |  |  |  |
| **Model 3** | **Fixed Effects** | **Coefficient Estimate** | **Standard Error** | | | **95% CI** | **Z - value** | **P - value** | **df** | **AIC Value** |
|  | Intercept | -2.5020 | 0.3298 | | |  | -7.587 |  | 494 | 664.0414 |
|  | Pattern | 1.3437 | 0.2679 | | |  | 5.015 | 5.30e-07 |  |  |
|  | Rainfall | 0.5467 | 0.2332 | | |  | 2.345 | 0.0191 |  |  |
|  | **Random Effects** | **Standard deviation of random intercepts** | | | |  |  |  |  |  |
|  | Transect | 0.1606 | | | |  |  |  |  |  |
|  | Position | 0.5190 | | | |  |  |  |  |  |
